# Risk factors for temporomandibular disorder: Binary logistic regression analysis

**DOI:** 10.4317/medoral.19434

**Published:** 2013-12-07

**Authors:** Bruno G. Magalhães, Stéphanie T. de-Sousa, Victor V C. de Mello, André C. da-Silva-Barbosa, Mariana P L. de-Assis-Morais, Márcia M V. Barbosa-Vasconcelos, Arnaldo F. Caldas-Júnior

**Affiliations:** 1 PhD student, Postgraduate Program in Dentistry, Universidade Federal de Pernambuco, Recife, Brazil; 2 Master’s student, Postgraduate Program in Dentistry, Universidade Federal de Pernambuco, Recife, Brazil; 3 Doctoral degree in Nutrition, Universidade Federal de Pernambuco, Brazil; Adjunct professor, Universidade Federal de Pernambuco, Recife, Brazil; 4 Postdoctoral degree in Epidemiology in Public Health, University of London, UK; Doctoral degree in Preventive and Social Dentistry, Universidade de Pernambuco, Brazil; Adjunct professor, Universidade Federal de Pernambuco, Recife, Brazil

## Abstract

Objectives: To analyze the influence of socioeconomic and demographic factors (gender, economic class, age and marital status) on the occurrence of temporomandibular disorder. Study Design: One hundred individuals from urban areas in the city of Recife (Brazil) registered at Family Health Units was examined using Axis I of the Research Diagnostic Criteria for Temporomandibular Disorders (RDC/TMD) which addresses myofascial pain and joint problems (disc displacement, arthralgia, osteoarthritis and oesteoarthrosis). The Brazilian Economic Classification Criteria (CCEB) was used for the collection of socioeconomic and demographic data. Then, it was categorized as Class A (high social class), Classes B/C (middle class) and Classes D/E (very poor social class). The results were analyzed using Pearson’s chi-square test for proportions, Fisher’s exact test, nonparametric Mann-Whitney test and Binary logistic regression analysis. Results: None of the participants belonged to Class A, 72% belonged to Classes B/C and 28% belonged to Classes D/E. The multivariate analysis revealed that participants from Classes D/E had a 4.35-fold greater chance of exhibiting myofascial pain and 11.3-fold greater chance of exhibiting joint problems. Conclusions: Poverty is a important condition to exhibit myofascial pain and joint problems.

** Key words:**Temporomandibular joint disorders, risk factors, prevalence.

## Introduction

Temporomandibular disorder (TMD) is a term used for a set of conditions that affect the temporomandibular joint and orofacial musculature (1,2). TMD can cause stiffness, joint noises, restricted mandibular movements and orofacial pain (3). Orofacial pain is an important factor directly associated with oral health-related qua-lity of life (4).

The prevalence rate of TMD is quite variable in the literature. Epidemiological studies estimate that 40 to 75% of the population worldwide exhibit at least one sign of TMD, such as joint noises, and 33% exhibit at least one symptom, such facial or joint pain (5-7). A number of studies report a higher prevalence rate in the female gender as well as peaks in adolescence and early adulthood (8-11).

There is consensus on the multifactor etiology of TMD. The literature reports possible risk factors, such as stress, hormonal factors, genetic factors, ethnicity, social status and gender. However, this field of knowledge remains obscure and well-designed studies is needed to allow greater clarification of this condition (12-17). Socioeconomic factors play an important role in health. Income, schooling, occupation, economic status and social inequalities can hinder access to health services, information and exams necessary for the diagnosis and treatment of diseases (1).

The aim of the present study was to analyze the influence of socioeconomic and demographic factors (gender, economic class, age and marital status) on the occurrence of temporomandibular disorder.

## Material and Methods

The present pilot study was carried out with a sample of 100 individuals aged 15 years or older from urban areas in the city of Recife (Brazil) registered at Family Health Units (FHU). No restrictions were made regarding gender or ethnicity. Multi-stage cluster sampling was performed to include the entire city. The selection of Basic Health Units was performed randomly by lots. To obtain the sample size for the principal study, we used a multi-stage sample technique, where we first used a cluster sampling to define the neighborhood in Health Districts, then a systematic sampling to choose the FHU, and for last 100 volunteers, were randomly selected among users of FHU.

Ethical approval for all stages was granted by the local research ethics committee (CAAE 05650512.9.0000.5208). All volunteers that agreed to be in the study signed the informed consent form.

The diagnosis of TMD was determined using Axis I of the Research Diagnostic Criteria for Temporomandibular Disorders (RDC/TMD) (18), which addresses myofascial pain and joint problems (disc displacement, arthralgia, osteoarthritis and oesteoarthrosis). Individuals diagnosed with at least one of these conditions were classified as having TMD. Four examiners underwent a training and calibration exercise for the administration of the RDC/TMD. Intra-examiner and inter-examiner agreement were determined using the Kappa statistic (K = 0.90 and 0.82, respectively).

The socioeconomic status was determined using the Brazilian Economic Classification Criterion of the Brazilian Association of Research Companies. This classification uses education level of the head of the household; number of radios at home; number of refrigerators; washing machines and color TVs; availability of drinking water and sewage, number of rooms in the home (especially the number of washrooms) and the number of cleaning personnel who work in the home. ABEP scores vary from zero (the poorest) to 46 (the richest). The scores were transformed into social class categories.

Scores from 0 to 7 correspond to class E, 8 to 13 (class D), 14 to 22 (class C), 23 to 34 (class B), 35 to 46 (class A) (ABEP 2013). In 2013 the Brazilian Association of Research Companies has changed the categorization. Then, the actual classification is Class A1 and A2 (high socioeconomic level), B1 and B2 (medium-high socioeconomic level), C1 and C2 (medium-low socioeconomic level) and D-E Class (as a unique class - poor socioeconomic level).

For the association analyses, marital status was dichotomized as “married” (respondents in a stable union, whether living together or not) and “not married” (widowed, divorced, separated and never married). Age was dichotomized as “≤ 30 years” and “> 30 years”, since the literature has pointed out 30 years as an age peak for RDC/TMD diagnoses in adults (19).

Statistical analysis

The Shapiro-Wilk test was employed to determine the distribution of the data (normal or non-normal). Ca-tegorical variables were analyzed using Pearson’s chi-square test for proportions and Fisher’s exact test for 2 x 2 contingency tables. Continuous variables were analyzed using the nonparametric Mann-Whitney test. To determine associations between the dependent and independent variables we did a binary logistic regression analysis and variables with a p-value < 0.05 remained in the final model. Odds Ratios (OR) and 95% confidence intervals (CI) were calculated. The SPSS 17.0 program was used for all statistical analyses.

## Results

One hundred individuals aged 15 to 70 years (mean: 34.76 ± 13.47 years; median: 32 years) participated in the present pilot study. The majority was over 30 years of age (57%) and 83% were women. More than half of the sample was not married (53%). Regarding economic class, none of the participants belonged to Class A, 72% belonged to Classes B/C and 28% belonged to Classes D/E.

Fourteen percent of the participants were diagnosed with myofascial pain; 26% were diagnosed with disc displacement and 18% were diagnosed with joint problems (arthralgia, osteoarthritis and osteoarthrosis). Economic class was significantly associated with a diagnosis of myofascial pain; a greater proportion of individuals with this symptom belonged to Classes D/E (28.6%). No statistically significant associations were found between myofascial pain and gender, age or marital status ( Table 1).

**Table 1:**
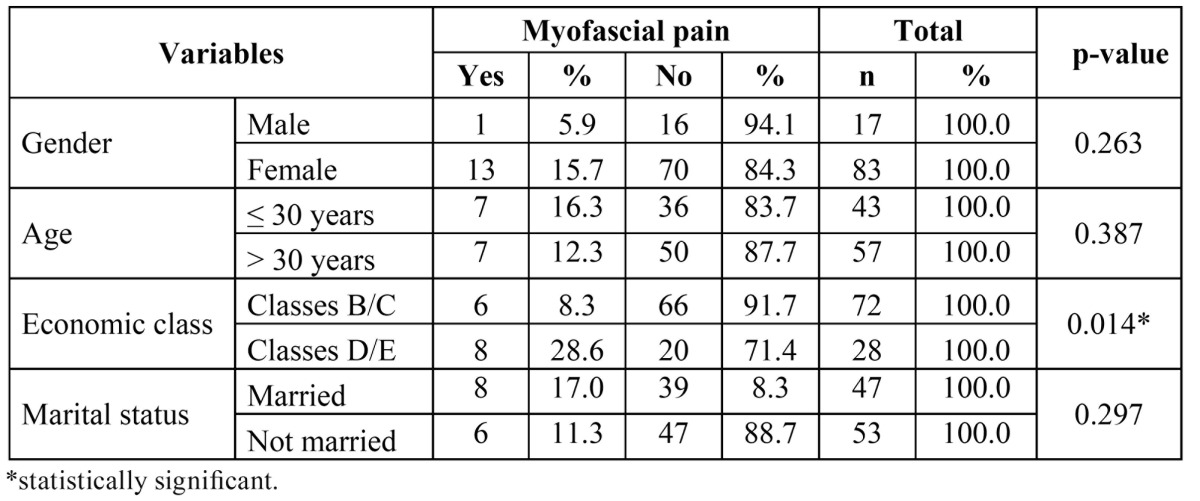
Distribution of participants regarding myofascial pain according to gender, age, economic class and marital status.

Fisher’s exact test revealed no statistically significant associations between disc displacement and gender, age, economic class or marital status ( Table 2).

**Table 2:**
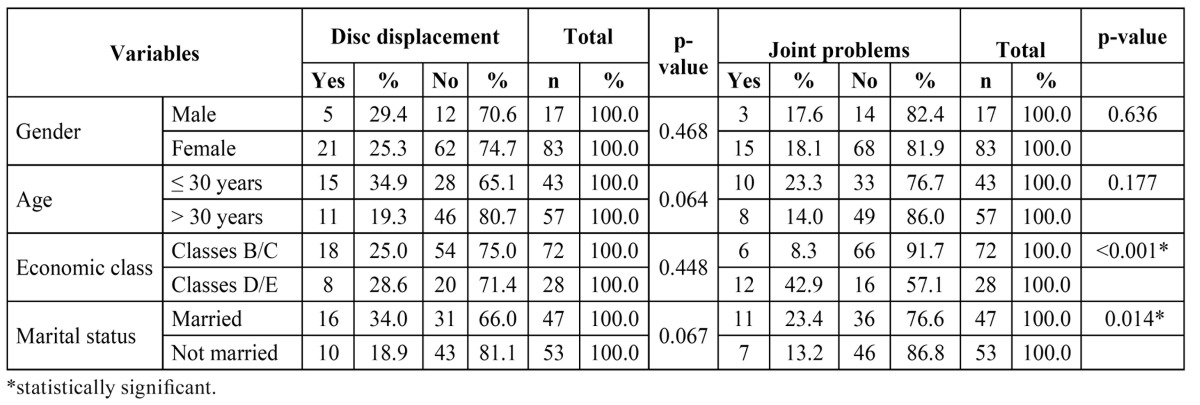
Distribution of participants regarding disc displacement and joint problems (arthralgia, osteoarthritis and osteoarthrosis) according to gender, age, economic class and marital status.

Economic class and marital status were significantly associated with joint problems (arthralgia, osteoarthritis and osteoarthrosis). The largest proportions of individuals with joint problems were married (23.4%) and belonged to economic classes D/E (42.9%). No statistically significant associations were found between joint problems and gender or age ( Table 2).

Multivariate analysis was performed using the binary logistic regression model to determine the OR for patients with and without TMD. Gender, age, economic class and marital status were incorporated into the model for myofascial pain. The Hosmer-Lemeshow test was used to determine the goodness of fit of the model, with a p-value > 0.05 demonstrating that the model fit the data. The multivariate analysis revealed that economic class was associated with a diagnosis of myofascial pain, as participants from Classes D/E had a 4.35-fold greater chance of exhibiting myofascial pain ( Table 3).

**Table 3:**
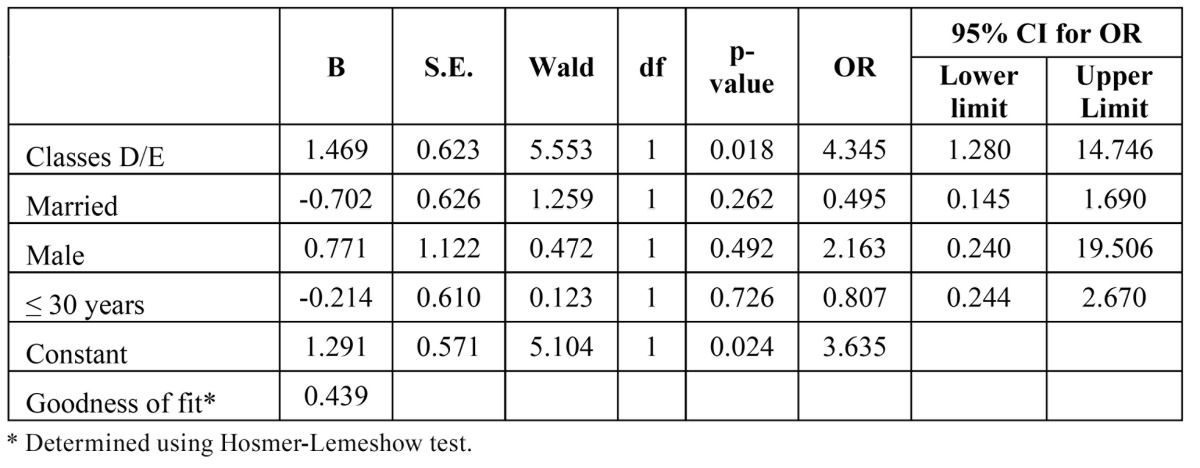
Final logistic regression model for myofascial pain according to economic class, marital status, gender and age.

The multivariate analysis revealed that social class and a diagnosis of myofascial pain were associated with joint problems. Individuals belonging to Classes D/E had an 11.3-fold greater chance of exhibiting joint problems ( Table 4).

**Table 4:**
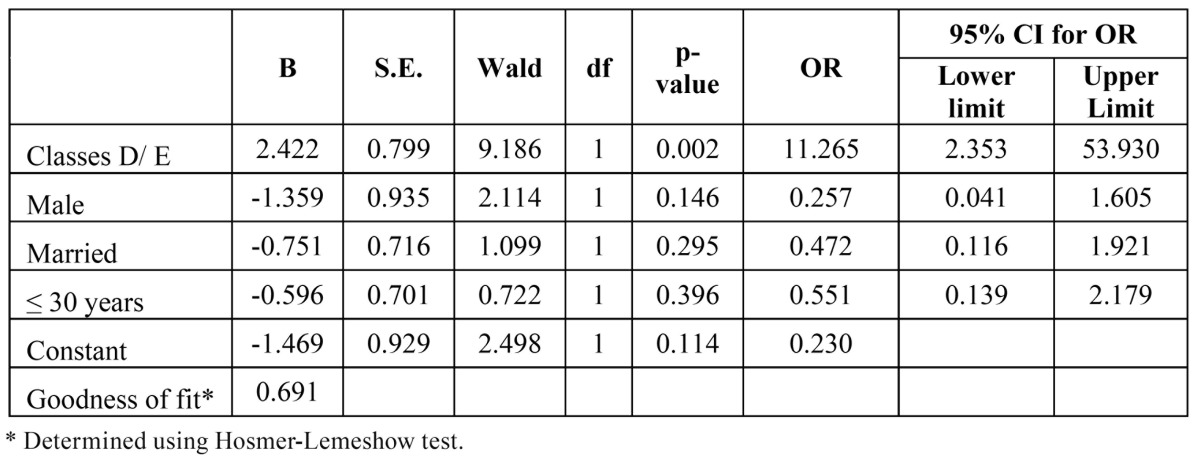
Final logistic regression model for joint problems according to economic class, gender, marital status and age.

## Discussion

Temporomandibular disorder is a widely studied, complex subject. However, a number of gaps in knowledge on this condition remain to be filled. The different etiologies discussed in the literature continue to be the subject of debate and disagreement among researchers and this lack of consensus has hampered the establishment of effective treatment protocols. Epidemiological studies are useful for the management of healthcare services by allowing the determination of the profile of a given population and assisting in the establishment of public policies aimed at controlling and eradicating adverse health conditions. The different prevalence rates described for TMD in the literature may be explained by the complexity and multifactor etiology of this disorder (1,20).

The prevalence of TMD in the present study (42%) was determined based on any symptom listed on Axis 1 of the RDC/TMD in a sample made up mostly of women aged 30 years or older. However, no significant association was found between TMD and gender in this study, which is in disagreement with findings described in previous studies (8). A number of theories have been put forth to explain the predominance of the female gender in cases of TMD, including the influence of biological, emotional and cultural factors (8,14). Among the biological factors which explain a greater association of TMD in women, the influence of genetic and hormonal factors have been mentioned in the literature (14).

The influence of socioeconomic factors on different health conditions is widely recognized. Individuals with higher incomes have greater access to information on health and preventive treatment, which can diminish the likelihood of disease progression. Such individuals are also less exposed to risk factors such as precarious housing, nutrient-poor foods, etc (1). In the present study, economic classes D and E (indicating lower economic status) were significantly associated with a greater prevalence of myofascial pain and joint problems (arthralgia, osteoarthritis and osteoarthrosis). One hypothesis for this finding would be exposure to risk factors such as precarious work conditions and food insecurity, which constitute stressors that may contribute to the development and perpetuation of TMD. Indeed, a number of studies have indicated stress and low socioeconomic status as important components of this disorder (8-10,21,22).

The results of the present study and a brief review of the literature demonstrate the influence of socioeconomic/demographic factors on TMD. However, these findings should be interpreted with caution, considering the size of the sample and the fact that the present investigation constitutes a pilot study, and it was a principal limitation of this study. Moreover, as the sampling procedure given for the pilot study was identical to the main study, the results probably will be the same.

This report describes the prevalence of TMD an how the RDC/TMD may continue to serve the function of guiding future research and, most importantly, serve as an evidence-based diagnostic and classification system to aid in the rational choice of clinical care for TMD sufferers around the world.

## Conclusion

The present study suggest that poverty is a important condition to exhibit myofascial pain and joint problems.
